# Increasing Sequence Search Sensitivity with Transitive Alignments

**DOI:** 10.1371/journal.pone.0054422

**Published:** 2013-02-14

**Authors:** Ketil Malde, Tomasz Furmanek

**Affiliations:** Institute of Marine Research, Bergen, Norway; University of Cyprus, Cyprus

## Abstract

Sequence alignment is an important bioinformatics tool for identifying homology, but searching against the full set of available sequences is likely to result in many hits to poorly annotated sequences providing very little information. Consequently, we often want alignments against a specific subset of sequences: for instance, we are looking for sequences from a particular species, sequences that have known 3d-structures, sequences that have a reliable (curated) function annotation, and so on. Although such subset databases are readily available, they only represent a small fraction of all sequences. Thus, the likelihood of finding close homologs for query sequences is smaller, and the alignments will in general have lower scores. This makes it difficult to distinguish hits to homologous sequences from random hits to unrelated sequences. Here, we propose a method that addresses this problem by first aligning query sequences against a large database representing the corpus of known sequences, and then constructing indirect (or *transitive*) alignments by combining the results with alignments from the large database against the desired target database. We compare the results to direct pairwise alignments, and show that our method gives us higher sensitivity alignments against the target database.

## Introduction

During the last decade, new developments in sequencing technology have resulted in lower costs and larger sequence volumes. Genome and transcriptome sequencing is now performed almost routinely, and the amount of publicly available sequence data has grown enormously. In contrast, correctly identifying the sequence by experimental means remains expensive, and as sequencing volumes continue to grow exponentially the proportion of sequences that can be analyzed in the lab is quickly diminishing. Thus, accurate and efficient computational methods for correct indentification and annotation of newly produced sequences remains an increasingly important challenge.

Typically, computational annotation involves searching for similar but known sequences using search tools like BLAST [1]. Putative function can then be derived by transferring annotation from matches deemed to be of sufficient reliability, typically using an alignment score threshold or BLAST 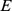-value. Although a large number of sequence data exists in public databases (as of March 2012, TrEMBL [2] contains close to twenty million proteins, and NCBI's RefSeq [3] almost fifteen million), only a few model organisms can be considered well studied, and most annotation is derived from homology or other automated means. Consequently, many sequences in these databases lack precise and detailed annotations.

In contrast, curated databases like SwissProt [2] generally provide much better annotations based on experimental study of the proteins in the database. The downside is limited proteome coverage, and although several thousand eukaryote species are represented in SwissProt [2], only 18 of them are represented with more than 1000 proteins.

A lack of close homologs tempts the researcher to use lower similarity thresholds, decreasing the reliability of matches and increasing the likelihood of false positives and incorrect annotations. This problem is exacerbated further when the species or process under study is phylogenetically remote from model organisms like human, mouse, or yeast.

Thus, the dilemma faced by the researcher seeking to identify novel sequences is the choice between *a*) a small, curated database like SwissProt, which would produce precise and detailed descriptions but where low similarity scores would mean that transferred annotations would not necessarily be accurate, or a large database like TrEMBL which would result in higher confidence alignments, but to proteins with less informative and possibly unreliable descriptions.

Here we propose an approach that combines the sensitivity of searching a large database with the detailed annotation from a curated database. This is achieved by calculating alignments against the curated database *indirectly*, combining hits against a comprehensive database with alignments between the comprehensive database and the curated one.

Our approach is inspired by algorithms for multiple sequence alignment (MSA), and in particular the *T-Coffee* [4] algorithm, which uses *triplets* of pairwise aligned sequences to increase overall consistency of the multiple alignments by avoiding early local misalignments. These triplets are similar to our transitive alignments, but in contrast to the MSA problem which is NP-complete and therefore must depend on heuristics [5], we limit our aim to producing pairwise alignments, and thus we are able to provide an optimal solution.

## Methods

In the following, we first define and describe the *composition* of two pairwise alignments to produce a *transitive alignment*. We then show how to combine a set of alignments into a consensus alignment, and discuss a scoring scheme and our current implementation.

### Composing alignments

We define an *alignment* of a pair of sequences as a function (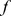, say) that maps positions in one sequence to positions in the other. In addition, we require two invariants, first that the mapping is *one-to-one*, that is, given positions 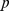 and 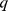,
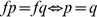
(1)


**Figure 1:**
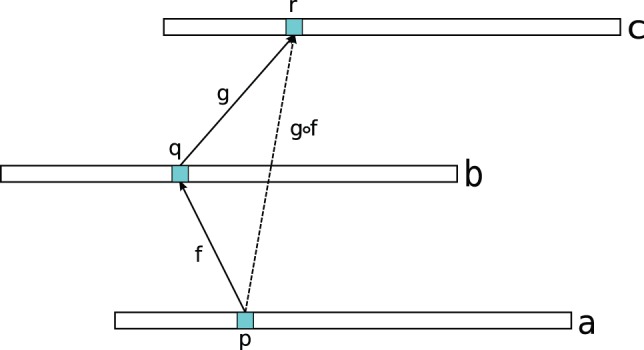
The alignments 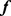 maps position 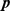 in 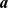 to position 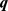 in 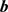 and the alignment 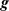 maps position 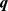 in 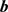 to 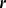 in 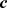. Thus, the composed alignment, 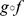, maps position 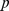 to position 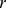.

It follows that the alignment invertible, i.e. there exists a mapping 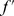 back from the second sequence to the first one. Second, the alignment is *monotonic* (or *colinear*), satisfying:
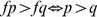
(2)


(Note that for or nucleotide sequences matches may be against the reverse-complement strand. In this case we represent target positions by their negation, and then the criterion still holds). Given an alignment 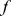 from a sequence 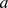 to a sequence 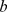, and an alignment 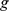 from 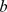 to a sequence 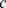, we define the *transitive alignment* to be the function composition 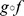, i.e. the mapping produced by first applying 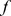 to each position in 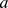 to get a coordinate in 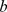, and then applying 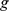 to get a coordinate in 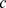. This is illustrated in Figure 1. It is easy to see that 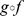 constitutes a valid alignment, as both the one-to-one property and the monotonicity is preserved.

Let 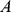 be a set of query sequences, 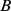 be a (large) set of intermediate sequences, and 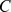 be a set of target sequences. Given a set of alignments 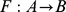, and 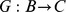, we can for each 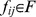 aligning a sequence 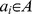 to a sequence 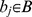 find the set of all 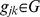 that aligns 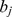 to some sequence 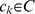. We can then construct the set of all transitive alignments 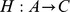 containing all alignments 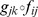. Note that for any 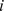 and 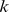 there may be several values of 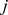, each representing one particular intermediate sequence. The set of transitive alignments 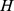 may therefore contain more than one alignment of sequences 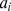 and 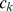.

### Scoring heuristic

The preceding description ignores some details for the sake of simplicity. In addition to providing a mapping between positions, alignments are also assumed to have an associated score. In order to calculate a score for transitive alignment, we first distribute the alignment score evenly to each pair of positions in the alignment. I.e., given an alignment of score 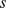 that aligns position pairs (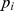,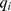) for 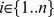, the alignment score for each position pair is 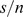. The composition of two alignments uses the minimum of the involved scores, so if, as in Figure 1, 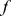 maps position 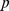 to position 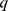 with score 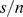 and 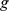 maps 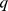 to 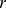 with score 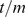, the transitive alignment 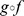 maps 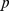 to 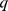 with a score of 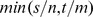.

For the entire alignment (e.g., for scoring the consensus alignments), the score is calculated as the sum of scores for each coordinate pair, recovering the original score if the alignment is constructed from a single alignment.

### Combining alignments

Given a set of (transitive) alignments 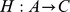, we can for any two sequences 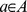 and 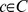 extract the subset 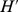 of alignments from 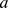 to 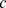. As these alignments are not necessarily consistent with each other, it remains to reconcile these in a single consensus alignment.

We formulate the problem as follows: given a set of scored alignments 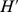 from 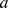 to 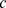, construct the combined alignment by choosing the subset of position pairs from all alignments in 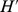 that maximizes the total sum of scores while satisfying Equations 1 and 2.

Let 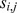 be the maximal alignment score in 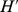 that aligns position 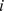 in sequence 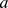 to position 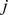 in sequence 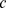. We can then calculate the total score 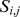 for aligning 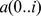 and 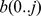 with the recurrence:
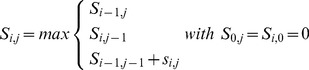
(3)


This recurrence is analogous to sequence alignment (without gap penalties), with a reward for aligning 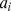 with 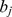 as the maximum alignment score linking these positions (i.e. 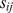), and we solve it similarly by dynamic programming.

## Results

To explore transitive alignments, we used UniRef 50 [6] as the intermediate database. Its entries are at most 50% similar on the sequence level, retaining a large variety of proteins while reducing the number of sequences to approximately 4 million – a more manageable number than the full UniProt database, but still about eight times the size of SwissProt, and encompassing a larger variety of proteins. Unless otherwise noted in the text, the following uses the NCBI BLAST suite version 2.2.25 using the default parameters.

### Protein structure identification

The SCOP database [7] classifies proteins based on structural alignments. Protein 3D structure is generally considered to be more conserved than protein sequence, and structural alignments is therefore able to identify homology in many cases where sequence comparison fails. At around 25–30% identity we find the *Twilight Zone*, where pairwise sequence alignments start to produce a considerable number of non-homologous hits [8].

**Figure 2:**
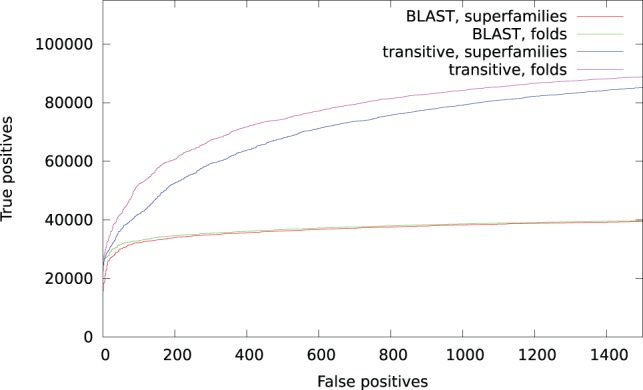
ROC curves of hits from SCOP 40 to itself. Hits to the same SCOP classification are considered true positives, hits with different classifications are considered false positives.

We downloaded SCOP 40, version 1.75A. This contains 11 211 proteins from SCOP with at most 40% identity, classified into SCOP's categories of class, fold, superfamily, and family. We used BLASTP to align this set of proteins against itself, and we also generated transitive alignments by using BLASTP to match the SCOP proteins to UniRef 50, and BLASTP alignments from UniRef 50 to SCOP 40, in both cases with an 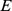-value threshold of 0.1. The results were sorted according to alignment score, and classified as true positives if the query and target had the same classification, false positives if they had different classification. The resulting ROC curves for the SCOP superfamily and fold levels are shown in Figure 2.

We see that for the same number of false positives, we are able to identify a higher number of true positives using transitive alignments. We also see that as we increase the number of false positives, transitive alignments gain true positives at a higher rate. Using direct BLAST with the default 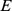-value threshold of 10, we identified a total of 47 908 false and 54 222 true positives at the superfamiliy level. Using transitive alignments with the same number of false positives, we identified 112 718 true positives, more than a twofold improvement. We also attempted the BLAST analysis specifying different substitution matrices, using BLOSUM-90 and BLOSUM-45 instead of the default BLOSUM-62. This did not have any substantial effect on the outcome, and BLOSUM-62 produced marginally better results than the alternatives.

It is also interesting to compare the results for superfamilies and folds. We see that using the looser criterion of folds only marginally increases the number of true positives for BLASTP, but that there is a substantial improvement for the transitive alignments. For instance, at 500 false positives, we find 36 243 true positives using BLAST at the superfamily level, with an additional 459 (1.3%) hits at the folds level. For transitive alignments, we identify 67 932 true positives at the superfamily level, increasing by 6 419 (9.4%) at the folds level.

### Transcript annotation

The salmon louse *Lepeophtheirus salmonis* is a small crustacean that is parasitic on salmonid fish. It is phylogenetically distant from most well-studied species, with copepods believed to have separated from insects more than 300 million years ago [9]. In addition, the salmon louse has a specialized life cycle, which adds to the challenge of identifying its proteins.

As part of the Salmon Louse Genome Project a set of 45 360 putative transcripts have been predicted *ab initio* from a draft genome assembly using Augustus [10]. In the following, we will investigate how transitive alignments can be used to annotate these sequences.

The input sequences were aligned against UniRef 50 using BLASTX and the UniRef 50 sequences were aligned against SwissProt using BLASTP, in both cases using an 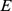-value threshold of 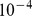. Transitive alignments were then calculated from these sets of BLAST hits, resulting in 23 457 transcripts receiving at least one alignment to a SwissProt sequence.

### Sensitivity

**Figure 3:**
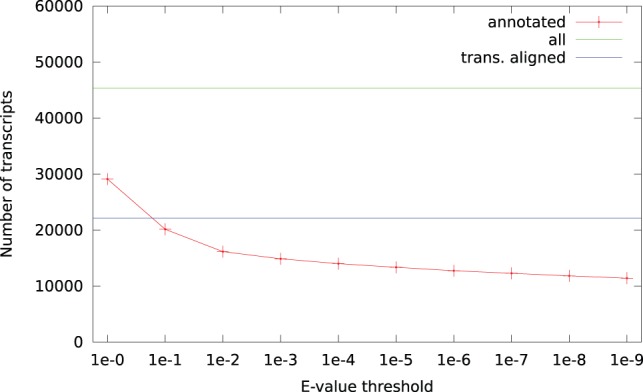
The number of transcripts aligned against the SwissProt database using increasing 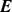-value threshold. For comparison, the total number of transcripts (45 360), and the number of transcripts annotated using transitive alignments with BLAST 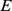-values of 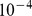 (22 162) are also shown.

In order to compare our approach to a traditional BLAST analysis, we aligned the putative transcripts directly against SwissProt using BLASTX. Using a maximum 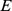-value of 1, we were able to align 29 137 (64%) transcripts, but as shown in Figure 3, the number decreases rapidly with stricter 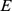-value thresholds, and a large fraction of transcript only have low confidence alignments. To get the same number of aligned transcripts as we found using transitive alignments requires an 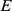-value threshold of 0.33.

**Figure 4:**
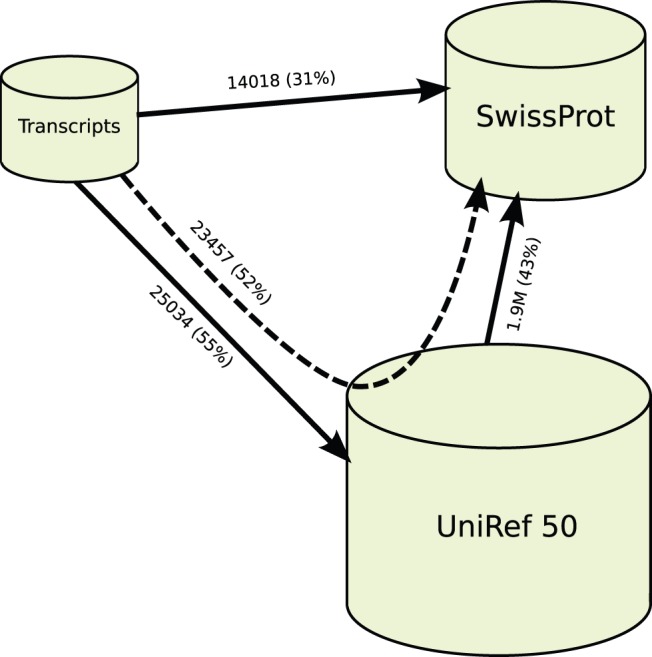
Sequence databases, and the number of query sequences with BLAST hits, using an 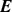-value threshold of 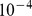. Numbers in parentheses indicate the percentage of query sequences with BLAST hits, transitive alignments are represented by the dashed arrow.

A direct alignment of the putative transcripts with an 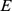-value threshold of 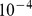 to SwissProt results in 14 018 (31%) of the sequences matching at least one protein sequence. Aligning the transcripts to UniRef 50 results in 25 034 (55%) sequences matching, and aligning UniRef 50 against SwissProt results in 1.9 million (43%) of the UniRef proteins being aligned. This is summarized in Figure 4. In light of these numbers, the number (23 457, or 52%) of transitively aligned sequences is high, and sequences in UniRef 50 that are aligned to a transcript also have a higher probability than average of matching a SwissProt sequence.

### Consistency

SwissProt and UniProt IDs (*entry names*) consist of two parts, a mnemonic protein ID (or *gene symbol*), and a mnemonic species ID. For instance, the protein CYAB_BORPE is the Cyclolysin secretion/processing ATP-binding protein gene (CYAB) found in the species *Bordetella pertussis* (BORPE). For each transcript, we extracted the gene symbol from the best SwissProt hit, and identified the position (rank) of the highest scoring transitive alignment to a protein with the same gene symbol (i.e., ignoring any differences in organism).

**Figure 5:**
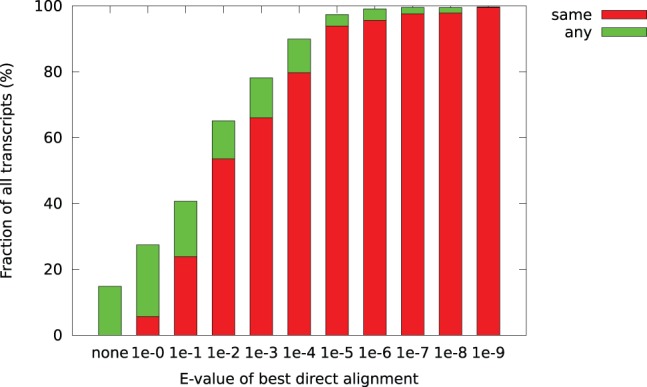
The fraction of all transcripts receiving an annotation using transitive alignment, grouped by e-value threshold of the transcript's best SwissProt match. Red bars indicate that the gene symbol of the best SwissProt match is also among the transitive annotations (see also Fig. 6), green bars that only different proteins were matched.

We partitioned the transcripts in categories based on the 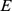-value for the best direct alignment to SwissProt. Figure 5 shows the fraction of the transcripts in each category receiving an annotation based on transitive alignments. We see that the likelihood of receiving a transitive annotation increases with the quality of the SwissProt alignment. For low 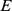-values, the consistency is low, and for the transcripts with only weak direct alignments (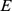-value 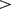
0.1), only 508 (5.7%) of them were transitively aligned to the same gene symbol as the direct alignment. Interestingly, a much larger number (1 957, 21.8%) received a different annotation.

**Figure 6:**
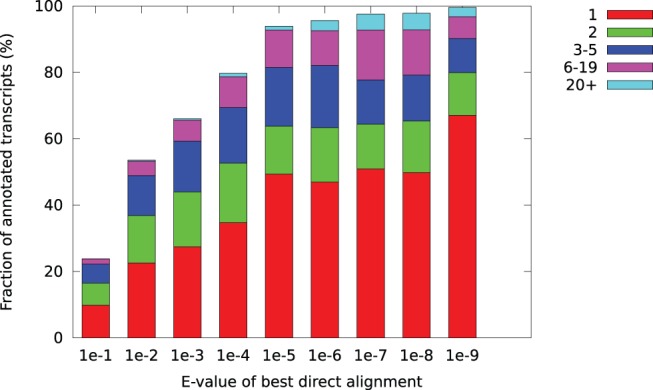
The fraction of transcripts with transitive alignments, categorized by the position (rank) of the best SwissProt hit among the transitive alignments. Proteins are considered the same if they have the same gene symbol.

A more detailed view on the consistency of the alignments is given in Figure 6. This shows the rank of the best SwissProt hit among the transitive alignments. We see that the best SwissProt hits are usually at or near the top of the transitive alignments, and that this is more pronounced for transcripts with good direct hits.

One important caveat is that gene symbols do not provide an unambiguous or even accurate way to identify orthologs, and for historical reasons, orthologous genes are often assigned different gene symbols. Also, members of closely related gene families can be difficult to distinguish, and will often have similar, but not identical, gene symbols. For instance, the transcripts g34 and g35 were annotated based on direct alignments as LCE and NAS4, respectively. Their transitive alignments assigned both to NAS15, which means that for the purposes of our analysis, both transcript were counted as a changed gene symbol. However, both the NASx family and LCE genes are metal-binding proteinases in the peptidase M12A family. A closer inspection of the genome revealed that g34 and g35 were indeed the same gene, and that they were incorrectly predicted to be separate transcripts due to a misassembly of the genome, where a short region had been erroneously duplicated in the scaffolding process.

### Improved alignments

**Figure 7:**
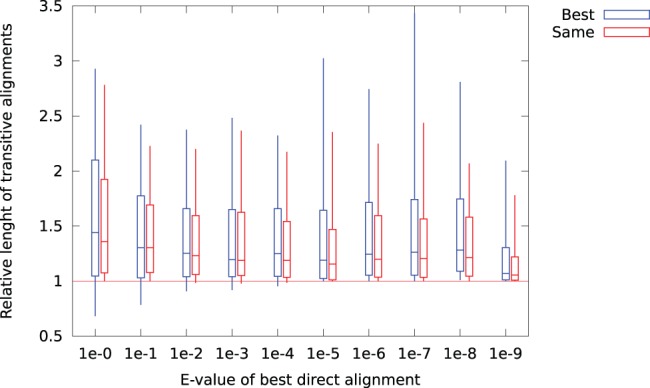
Alignments lengths resulting from transitive alignments divided by the corresponding score for the best direct alignment. The plot shows median, quartiles, and 10- and 90-percentile length ratios, for the best transitive alignment (blue), and for the best transitive alignment with the same gene symbol as the best direct alignment (red).

**Figure 8:**
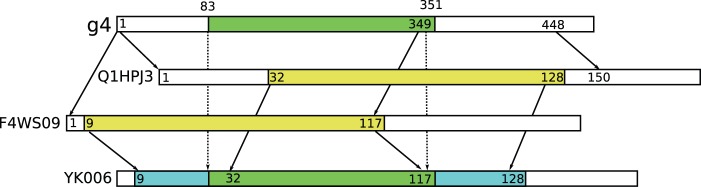
Using BLASTX to align the transcript (g4) to SwissProt identifies a short match (green) against YK006. BLASTX searches against UniRef 50 produces alignments (yellow) to proteins Q1HPJ3 and F4WS09. Both of these also match YK006, and these are used to construct the transitive alignment (blue) that covers almost the complete transcript. (For simplicity, other intermediate hits in UniRef 50 are omitted).

With a more sensitive local alignment algorithm, we expect to see longer alignments in the case where the correct homolog is identified. For each transcript, we calculated the ratio of transitive alignment length to the direct alignment length, both for the highest scoring transitive alignment, and for the same target sequence as the best direct alignment. The results are shown in Figure 7. We see that for all categories, more than 75% of the sequences produce longer transitive alignments than direct alignments. Here also, we see the tendency of lower improvements for the best alignment category. An example where a longer alignment is discovered using transitive alignments is shown in Figure 8. Here, the annotation for one transcript is extended through the use of several intermediate matches, resulting in a transitive alignment of length 145 amino acids, in contrast to the direct alignment length of 89 amino acids.

### Pfam domains and novel annotations

**Figure 9:**
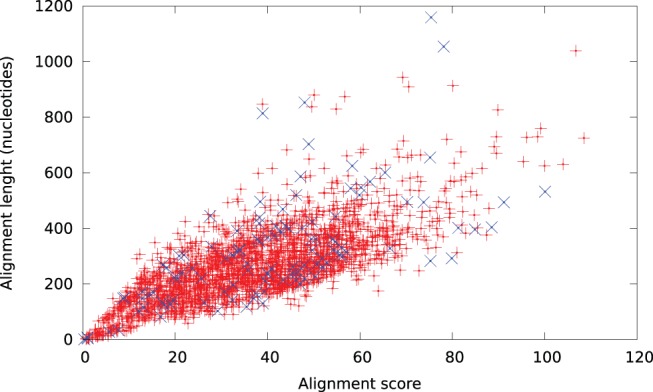
Length and score of transitive alignments for transcripts that had no direct BLAST match. Although not directly comparable, direct alignments with an 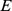-value of 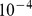 have alignment scores averaging 44.4. Data points marked with as 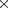 represent transcripts with an identified Pfam domain.

Of the transcripts, 16 223 (35.8%) had no direct BLAST hits to SwissProt. Using transitive alignments, we were able to identify an alignment for 2405 of these. The distribution of alignment scores and lengths are given in Figure 9. As expected, most alignments are short and with low scores, but there are also several transcripts with long or high scoring alignments.

SwissProt records are, among other information, annotated with identified protein domains. We therefore compared the transitive alignments to the results of using HMMER with Pfam to identify domains in the sequences. Of the full set of transcripts, HMMER was able to annotate 13 393 (29.5%) with a Pfam domain. (If we limit the 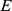-value to 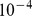 the number of transcripts with an identified domain is 12 985, or 28.6%). For each of the transcripts we examined the SwissProt record of the best transitive alignment hit. We found that for 12 727 (95.0%) transcripts, the highest scoring protein was annotated with the domain identified using HMMER.

Of the transcripts without direct BLAST hits, only 117 (4.7%) were annotated with a Pfam domain annotation using HMMER, these are shown separately in Figure 9. Examining the SwissProt records of the highest scoring transitive alignment, we find that 93 (79.5%) of them are annotated with the same Pfam domain as HMMER identifies.

The most commonly occurring domains in this set, and the corresponding transitive alignments, are listed in Table 1. Here we see that our annotations are quite consistent with the identified domains, for instance: all the THAP domains occur in sequences identified as THAPx variants; the Transposase_1 and HTH_Tnp_Tc3_2 (helix-turn-helix) domains are identified in sequences all annotated as transposases; and POGK, POGZ, TIDG6, and JERKL are all annotated with the DDE_1 endonuclease domain in UniProt.

**Table 1:** The most frequent Pfam motifs identified in the novel transcripts.

Pfam Motif	SwissProt hit	Count
Chitin_bind_3	OR92A_DROME	2
CUE	TC1A_CAEEL	1
	TCB2_CAEBR	1
DDE_Tnp_IS1595	Y132A_HAEIN	2
DUF227	DHS27_CAEEL	2
DUF659	CGBP1_MOUSE	2
MTTB	MTTB_DESHY	2
Peptidase_A17	POL4_DROME	1
	TAAR4_MOUSE	1
RVP	YRD6_CAEEL	2
rve	YRD6_CAEEL	3
RVT_1	LIN1_NYCCO	1
	PO11_NASVI	1
	RTJK_DROME	1
Zona_pellucida	CUT1_CAEEL	2
	ELDP2_LOTGI	1
DDE_1	POGK_HUMAN	2
	POGK_MOUSE	1
	POGZ_MOUSE	1
	TIGD6_HUMAN	1
	JERKL_MOUSE	1
THAP	HAP2_HUMAN	3
	THAP4_BOVIN	2
	THAP2_MOUSE	1
	THAP1_DANRE	1
	THAP9_HUMAN	1
Transposase_1	MOS1T_DROMA	11
	SETMR_HUMAN	2
HTH_Tnp_Tc3_2	TCB1_CAEBR	5
	TCB2_CAEBR	4
	TC1A_CAEEL	3
	TC3A_CAEEL	3
	SETMR_HUMAN	1
	MOS1T_DROMA	1

**Figure 10:**
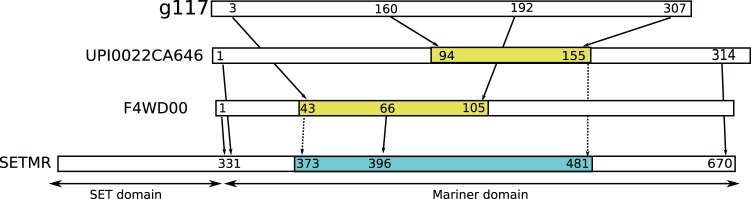
The g117 transcript had no significant BLASTX hits to SwissProt, nor any annotated Pfam domains. Again, by using hits against two UniRef 50 sequences, we can calculate a transitive alignment that covers the entire transcript sequence. Interestingly, the SETMR gene is primate specific, and consists of a Histone-lysine N-methyl transferase (SET domain, EC 2.1.1.43) with an inserted transposase (Hsmar1, EC 3.1), and it also has one isoform without the transposase. The transitive alignment of g117 is against the transposase domain, and the alignments of the intermediate proteins also match this part almost exactly, so in all likelihood, the SET domain is irrelevant for g117. Nevertheless, the annotation of F4WD00 in UniProt is as a methyl transferase. An annotation based on this would thus be incorrect, illustrating the dangers of using an uncurated database.

An example of a novel annotation is shown in Figure 10, where the transcript is aligned via two different UniRef 50 sequences. Both sequences map consistently to a SwissProt sequence, but with little overlap. The transitive alignment thus covers the whole transcript, and it can be unambiguously annotated as a fragment of a gene similar to mariner transposase [11].

## Discussion

There are now vast amounts of easily available sequence data, and although large resources are committed to curation and experimental validation, the proportion of uncurated data is large and growing. As the raw data becomes cheaper to produce, we need automatic methods that can exploit the raw data directly. The method we present here use uncurated sequence data to provide a context for sequence alignments, and is able use this context to construct alignments with higher sensitivity than direct, pairwise alignments.

As a typical transcript annotation experiment, we aligned transcripts to SwissProt, and found that using transitive alignments with a relatively conservative 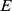-value threshold identified alignments for a larger fraction of the query sequences than direct alignments with similar parameters. Also, the alignments identified transitively are substantially longer, indicating higher sensitivity. We also see that we are able to find identified Pfam domains from transitively aligned protein matches.

Perhaps the most significant result is the identification of SCOP folds and superfamilies. Here we see that not only does transitive alignment identify the known SCOP relationships more accurately than direct BLAST, but while BLAST mainly find superfamily relationships, transitive alignments are also able to identify many relationships at the more distant fold level. This is perhaps the clearest evidence that our method offers substantial improvement over direct BLAST.

### Related work

Perhaps the best known tool for identifying distantly related homologs is PSI-BLAST [1]. It uses the result of an initial BLASTP query against a database to build a position-specific scoring matrix (PSSM), which is then used to search the database again to identify more matching sequences, and this process is then iterated.

Similar to the PSSMs generated by PSI-BLAST, it is possible to identify functionally related proteins through shared motifs or domains, often described using hidden Markov models. Typically, motif or domain databases (e.g. Pfam [12] or SMART [13]) are derived from collections of proteins, and used with search programs like HMMER [14] to search the candidate sequences.

Although these tools share the goal of identifying homology, they differ from our approach in several important respects. First, although we also make use of multiple sequences to strengthen the results, our aim is to identify individual homologs. Thus, our method serves as a drop-in replacement for regular BLAST, and the method is simpler both to understand and to use as it avoids stochastic models of sequences or sequence fragments. In contrast to PSI-BLAST, our method can be used to annotate nucleotide query sequences directly, and where PSI-BLAST results in a cluster of related sequences, our method results in a set of individual sequences in a specific target database.

Combining or post-processing pairwise BLAST results is not a new approach, and e.g. taking the best alignment score between groups of sequences has been shown to identify protein family relationships with a higher sensitivity than using stochastic models, at least for small groups of sequences [15]. Our method differs in that it does not depend on any *a priori* grouping or classification, and that it produces the alignment of sequence positions, and not just a similarity score.

### Future Directions

In our analyses we have kept the intermediate and target databases separate. This serves to distinguish the effect of transitive alignments from direct alignments, but will lead to inferior results in some cases when the sequences in the intermediate database match neither query or target sequence well. In practice, this is easily avoided by merging the seqeunces from the target database into the intermediate database.

In our current experiment, we have used BLAST-based transitive alignments from nucleotide sequences to proteins, and from proteins to proteins. It is of course possible to use different combinations of sequence databases, or to use different alignment software. It is also possible to extend the chain of databases by introducing more intermediate databases.

Similarly, the method is not limited to traditional sequence alignments. For instance, SwissProt contains annotated features for many of its sequences, and one could extract sequence-to-feature “alignments” to identify conserved domains. Functional annotation with Gene Ontology terms [16] is often derived from protein alignments [17], but as is illustrated by the example in Figure 10, this runs the risk of one conserved domain leading to an incorrect annotation with terms belonging to a different domain that happens to occur on the same sequence in the database.

Here we have used a simplistic scoring model for an alignment, distributing BLAST scores uniformly over the aligned region, and summing the scores for all aligned position pairs. This scheme has the property that it preserves the original BLAST score, but other schemes are certainly conceivable. The consensus calculation could for instance be modified to use affine gap penalties, and differentiate between global and local alignments. We have not studied transitive alignment scores in detail, and consequently, the alignment score is not interpreted beyond ranking alignments. More work is needed before we can give alignment scores any meaningful interpretation, for instance in terms of 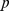
- or 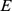-values.

## Conclusion

The choice of databases to use for sequence alignment is often a compromise between accuracy and precision. By using transitive alignments, we can leverage searches against large databases that give high sensitivity and produce stronger hits against a curated database than one would get directly, and thus make this compromise less severe.

The method is conceptually simple, and relies only on standard alignment software and sequence databases. Addition of new sequences to the databases and improvements in sensitivity in the alignment software (e.g., [18]–[20]) will therefore improve the results. Also, as the output is similar to BLAST output, transitive alignments can be used directly in existing analysis pipelines. In contrast to using precalculated patterns, motifs or classes, transitive alignments rely only on sequence databases. We therefore believe transitive alignments provides a useful and easy to use method, and a valuable addition to the molecular biology toolset.

### Availability

We have implemented transitive alignments in Haskell, using version 7.4.1 of the GHC compiler. The source code is available under a GPL license from http://malde.org/ketil/biohaskell/transalign.
